# No unfavorable effects on the menstruation recovery of early postoperative hypoprolactinemia after transsphenoidal surgery in patients with lactotroph pituitary neuroendocrine tumor

**DOI:** 10.1186/s13104-024-06866-w

**Published:** 2024-07-30

**Authors:** Tomohisa Ishida, Tomohiro Kawaguchi, Yoshikazu Ogawa, Teiji Tominaga, Hidenori Endo

**Affiliations:** 1 Department of Neurosurgery, Kohnan Hospital, 4-20-1 Nagamachi minami, Taihaku-ku, Sendai, Miyagi 982-8523 Japan; 2 Department of Neurosurgery, Tohoku University Graduate School of Medicine, Sendai, Miyagi 980-8574 Japan; 3 Department of Neurosurgery, Senseki Hospital, Higashimatsushima, Miyagi 981-0501 Japan

**Keywords:** Endoscopic transsphenoidal surgery, Hyperprolactinemia, Hypoprolactinemia, Prolactin, Regular menstruation restoration, Endocrinological remission

## Abstract

**Objective:**

Transsphenoidal surgery for lactotroph pituitary neuroendocrine tumor (PitNET) lowers serum prolactin concentrations, occasionally below the normal range. However, the clinical significance of postoperative hypoprolactinemia is still unclear. In this study, we retrospectively reviewed the female patients with lactotroph PitNET who were treated with transsphenoidal surgery to elucidate the influence of postoperative hypoprolactinemia on regular menstruation restoration and endocrinological remission.

**Results:**

The serum prolactin levels in all thirty three participating females had decreased following surgery. Serum prolactin levels in seven patients had decreased below the lower limit of normal ranges (hypoproactinemia group) and in the remaining twenty six patients, it was within the normal range (non-hypoproractinemia group). In hypoprolactinemia group, regular menstruation was restored in all patients with only lactotroph axis deficiency. Nine patients from the non-hypoprolactinemia group experienced re-elevation of serum prolactin concentration (27%). No patient in hypoprolactinemia group experienced the relapse of hyperprolactinemia. These data suggest that early postoperative hypoprolactinemia after transsphenoidal surgery for lactotroph PitNET is not only a good predictive factor for endocrinological remission but also no unfavorable effects on regular menstruation restoration.

## Introduction

Lactotroph pituitary neuroendocrine Tumor (PitNET) is the most common functional pituitary tumor [1]. In female patients, a high concentration of serum prolactin affects the hypothalamic-pituitary ovarian system, causing insufficient folliculogenesis and oocyte maturation, in which in turn results in oligomenorrhea or amenorrhea and/or infertility [2, 3]. Transsphenoidal surgery can correct the hyperprolactinemic state by reducing the tumor mass, however, occasionally an excessive decrease in prolactin levels below the normal ranges has been observed. The clinical significance of postoperative hypoprolactinemia, especially the influence on the reproductive system, is still unclear.

The present study was a retrospective analysis aimed at clarifying the relationship between postoperative serum prolactin concentration and endocrinological outcome in female patients suffering from lactotroph PitNET. Concentration of serum prolactin, that is lower than the normal ranges might not be associated with prolonged menstrual disturbance.

## Patients and methods

### Patients

We retrospectively reviewed consecutive premenopausal female patients with lactotroph PitNET, who had presented with menstrual disturbance and had undergone surgical resection in Kohnan hospital between June 2015 and October 2022. Surgical intervention was advised for patients who were either resistant to or had a poor tolerance of medical therapy. Surgery was also performed for patients who preferred surgery rather than long-term medical therapy. We closely discussed about the advantage and disadvantage of both treatments, these patients understood and chose surgical treatment by themselves. Transsphenoidal tumor removal was performed on all patients under general anesthesia. Extracapsular resection from the pituitary gland was performed in all cases. At the end of removal, alcohol fixation for the surgical cavity was performed in case without evidence of intraoperative CSF leak. During the study period, surgery was performed by two neurosurgeons (Y.O. and T.K.) based on a consistent treatment concept originally developed by senior neurosurgeon Y.O. Surgical procedure was performed under both microscopic and endoscopic observations before 2020. A complete endoscopic procedure was adopted in 2021. Informed consent was obtained from each patient or guardian on admission and prior to the surgery and all methods were performed in accordance with the STROBE guidelines [4].

### Patient characteristics and data collection

The following variables were recorded in a database and analyzed: Age, sex, and maximum diameter of the tumors. A tumor with a diameter less than 10 mm was defined as a smaller tumor, while a tumor larger than 10 mm was termed a larger tumor. Histological diagnosis was performed in accordance with the WHO 2022 classification using hematoxylin and eosin staining as well as immunohistochemistry [1, 5]. Serum concentrations of prolactin and other basal anterior pituitary hormones (GH, TSH, LH, FSH, and ACTH) and their target hormones (free tri-iodothyronine, free thyroxine, insulin-like growth factor 1, and cortisol) were measured before surgery, seven days following surgery, and three months following surgery. Thereafter, serum prolactin levels were measured after every six months. 4.91 ng/ml–29.32 ng/ml was the normal ranges of serum prolactin concentration in premenopausal women observed in our hospital. Hypoprolactinemia is defined as prolactin levels below the normal ranges. Patients were divided into two groups based on serum prolactin concentrations measured seven days after surgery (hypoproactinemia group and non-hypoproractinemia group). All endocrinological evaluations were performed by specialist in endocrinology.

### Statistical analysis

Categorical variables were expressed as absolute numbers and percentages. Serum prolactin concentrations were expressed in terms of means and standard deviations. The Kaplan-Meier method was used to estimate the probability of relapse following initial normalization. Data were censored on December 1, 2022. Patients experiencing postoperative hyperprolactinemia were censored at the date of the measurement. Probability values < 0.05 were considered statistically significant. EZR (Easy R) software were used for statistical analyses [6].

## Results

### Basic characteristics

All thirty three patients included in the study exhibited improved serum prolactin concentrations at POD7 (post operative day 7) and had been followed up for median 29 months (range 4–84 months). Median age of the participants was 26 years (range 15 to 43 years). Among the thirty three patients, eighteen had tumors that were less than 10 mm in diameter, and the tumors of the remaining patients ranged from10 to 25 mm in diameters. Two patients were accompanied with preoperative visual disturbance. Preoperative hypopituitarism was not observed. Preoperative prolactin concentrations in the participants ranged from 60.3 to 1800 ng/ml. Postoperatively, hypoprolactinemia was detected in seven patients (hypoprolactinemia group), while the remaining twenty six patients exhibited serum prolactin concentration that was within the normal ranges at POD7, and had not experienced hypoprolactinemia during the follow up period (non-hypoprolactinemia group). The basic characteristics of patients, including age, the ratio of smaller tumor and larger tumor, and preoperative serum prolactin concentrations were not statistically different between the two groups, as evident from Table 1.

**Table 1:** Characteristics of enrolled patients

	Hypoprolactinemia (*n* = 7)	Nonhypoprolactinemia (*n* = 26)	*P* value
Age, range (median)	24 (16–32)	27 (15–43)	0.313
Smaller tumor: Larger tumor	3:4	14:12	1.000
Previous medical treatment	0 (0%)	3 (12%)	1.000
Serum prolactin concentration mean ± SD (ng/ml)	215.1 ± 123.7	242.4 ± 332.7	0.415
Gross total removal	7 (100%)	26 (100%)	1.000
Alcohol fixation	6 (86%)	23 (88%)	0.282
Recovery of regular menstruation	5 (71%)	26 (100%)	0.0398
Relapse of hyperprolactinemia	0 (0%)	9 (35%)	0.149

SD: Standard deviation

### The restoration of regular menstruation

During the follow up period all patients with only lactotroph axis deficiency in hypoprolactinemia group, reported spontaneous restoration of regular menstruation. The remaining two patients suffered from multiple axis deficiencies as lactotroph, gonadotroph and corticotroph axes, and menstruation was restored after multiple hormone replacement (6.1% of all patients). The detailed characteristics of hypoprolactinemia group are depicted in Table 2.

**Table 2:** Characteristics of patients with hypoprolactinemia

No	Age	Diamaer (mm)	Knosp grade	Suprasellar extension	Pretreatment	Pathology	Perioperative complication	Serum ploractine concentration (ng/ml)				Duration of F/U (Month)	Multiple axes deficit	Recovery of normal menstruation
								Preoperative	POD7	POD30	Last F/U			
1	21	15 × 12 × 14	0	No	No	Lactotroph PitNET	None	627	0.58	16.3	10.9	21	No	Yes
2	24	12 × 9 × 8	0	No	No	Lactotroph PitNET	None	398	2.91	9.55	12.4	15	No	Yes
3	27	6 × 5 × 3	0	No	No	Lactotroph PitNET	transient SIADH	94.5	3.08	7.19	5.54	15	No	Yes
4	32	8 × 5 × 6	0	No	No	Lactotroph PitNET	None	100.1	3.04	5.50	9.80	60	No	Yes
5	16	7 × 8 × 9	0	No	No	Lactotroph PitNET	None	383	1.25	3.63	3.97	29	No	Yes
6	20	11 × 11 × 11	1	No	No	Lactotroph PitNET	transient rhinorrhea	202	0.39	2.0	1.40	31	Yes	No
7	31	7 × 7 × 5	0	No	No	Lactotroph PitNET	transient DI & SIADH	118	1.12	0.62	0.80	13	Yes	No

DI: diabetes insipidus, F/U: follow up, Pit NET: pituitary neuroendocrine tumor, POD: postoperative day, SIADH: syndrome of inappropriate secretion of antidiuretic hormone

On the other hand, regular menstruation was restored in all patients from the non-hypoprolactinemia group.

### Postoperative serum prolactin concentrations

**Fig. 1:**
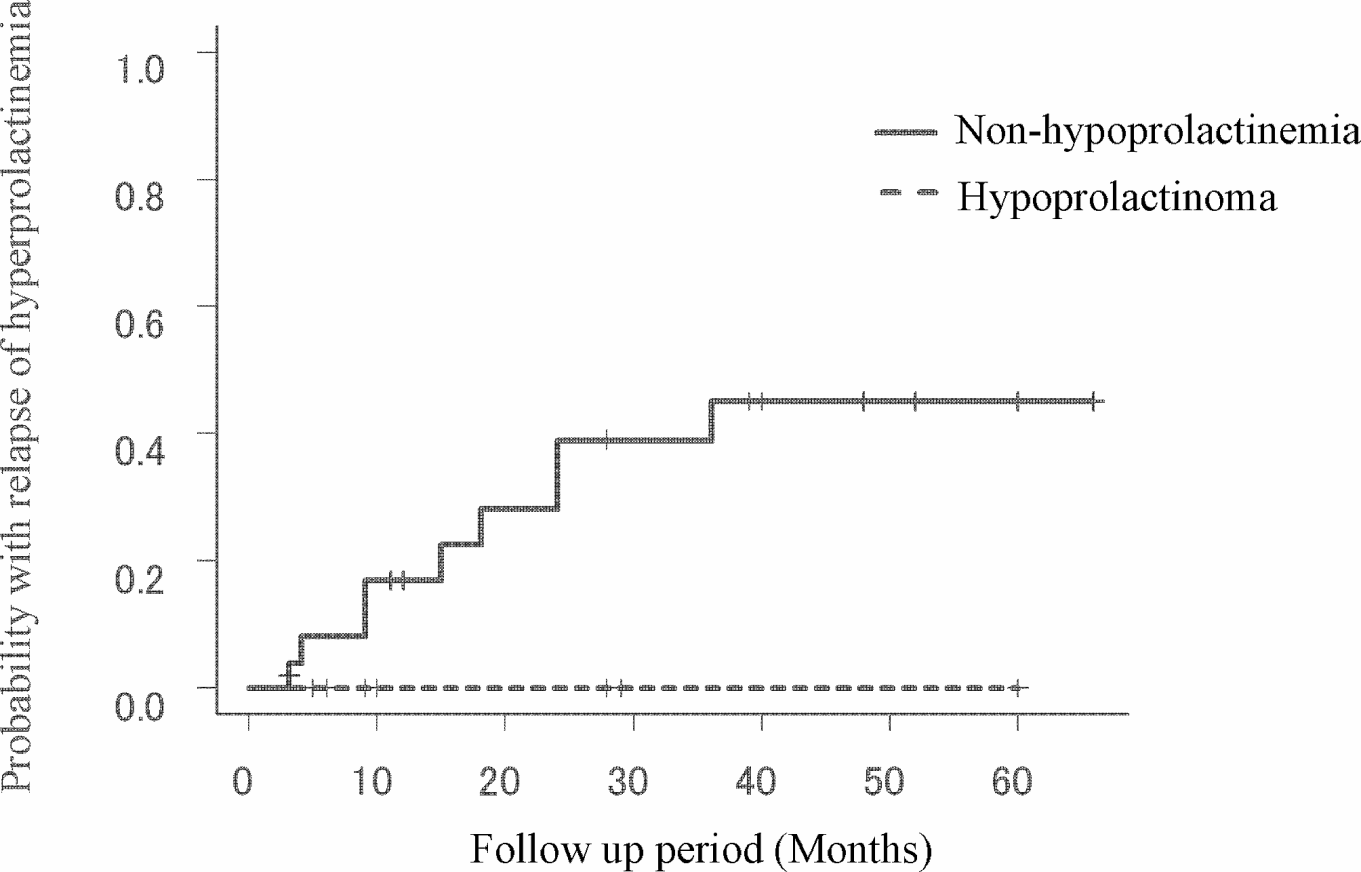
Kaplan Meier curve for determining the probability of hyperprolactinemia following initial normalization. The Kaplan-Meier curve demonstrates the probability of hyperprolactinemia following initial normalization in hypoprolactinemia group as well as in the non-hypoprolactinemia group. No patients with hypoprolactinemia experienced the relapse of hyperprolactinemia, but no statistical significance was observed (*P* = 0.178)

No patient from the hypoprolactinemia group experienced relapse of hyperprolactinemia during the follow up period. All patients from the non-hypoprolactinemia group, had normal serum prolactin concentrations on POD7 (7.03 to 25.3 ng/ml). However, the serum prolactin concentrations increased in nine out of the twenty six patients (27%), following POD7. Three out of nine patients were larger tumor, and the remaining were smaller tumor. For five out of nine patients were subjected to additional treatment such as dopamine agonists or gamma knife radiosurgery, which resulted in lowered serum prolactin concentrations. Remaining four o patients, due to slightly elevated serum prolactin concentrations (36.6 to 46.1 ng/ml) and normal menstruation cycle, observation without additional treatment was decided. Probability of hyperprolactinemia relapse in the follow up period that has been determined using the Kaplan-Meier method has been depicted in Fig. 1.

## Discussion

This study investigated the clinical significance of postoperative hypoprolactinemia in patients with lactotroph PitNET. The results demonstrated that early postoperative hypoprolactinemia did not adversely affect the restoration of regular menstruation, thereby maintaining endocrinological remission. To the best of our knowledge, this is the first study that has focused postoperative hypoprolactinemia on the menstruation recovery, and has many implications for the definition of successful treatment outcomes [7].

Generally, hypoprolactinemia is considered to be one of the causes of infertility affecting both women and men [8]. In women, low prolactin levels can inhibit formation and functioning of the corpus luteum, thereby inducing ovarian dysfunction [9]. One of the causes of hypoprolactinemia is anterior pituitary gland dysfunction resulting from pituitary lesions and autoimmune disease. Pituitary surgery could postoperatively result in the induction of new pituitary insufficiencies, as evident from the 6.1% incidence of multiple hormone deficiencies observed in this study. However, our results indicated that postoperative hypoprolactinemia did not adversely affect restoration of menstruation. All five patients with only lactotroph axis deficiency included in our study demonstrated early restoration of menstruation, postoperatively. This finding has not been documented in previous literatures.

The probability of relapse of hyperprolactinemia following initial normalization ranges from 17 to 50% for microadenomas and from 20 to 80% for macroadenomas [10–15]. Early postoperative prolactin levels might be significant with respect to long-term remission, some studies do recommend lower levels of serum prolactin concentrations [16–20]. In this context, our results are in alignment with the previous studies. Postoperative hypoprolactinemia could serve as a good marker for predicting prolonged endocrinological remission.

The exact reasons why immediate postoperative hypoprolactinemia does not influence on restoration of regular menstruation are unknown. The extent of tumor resection could be one possible factor. Prolactin levels measured immediately following resection surgery correspond to the existence of residual tumor, and an intensive resection, including removal of a layer of normal pituitary gland at the outer edge of the pseudocapsule is recommended to ensure complete excision [20–22]. Larger extent of tumor resection could possibly cause a larger damage of the anterior pituitary gland. The influence of the prolactin inhibiting factor could also play a role in the postoperative hypoprolactinemia. The secretion of prolactin is mainly regulated by inhibitory control by hypothalamic dopamine and is regulated in a negative feedback manner with prolactin itself, known as short-loop feedback system [2]. High concentrations of serum prolactin activates this afferent signal. Immediately following the transsphenoidal surgery for lactotroph PitNET with the rapid normalization of hyperprolactinemia state, prolactin inhibiting factor might be present in relatively high concentrations, resulting in excessive suppression of prolactin secretion.

## Limitations

The present study has several limitations. The number of patients is relatively small, since most patients with prolactinomas are treated only with medicines. The follow up period for patients with hypoprolactinemia was relatively short. More cases and longer follow up period are required for better understanding of hypoprolactinemia. Another limitation is the hypopituitarism caused by the pituitary surgery. Previous studies have mentioned a 22–55% incidence of new hypopituitarism in patients with PitNET [10, 23, 24]. Anterior pituitary dysfunction involving gonadotropin and thyroid hormone can also induce menstrual disturbances [18]. Although the incidence is lower than previously reported, two patients from our study (6.1%) suffered multiple anterior pituitary axes deficiency other than lactotroph, and additional hormone replacement therapy was required. The fact that hypopituitarism was observed only in hypoprolactinemia group, is indicating that more aggressive dissection for lowering prolactin level is potential risk of hypopituitarism. Prevention of deficiency involving axes other than the lactotroph is vital.

## Conclusion

Hypoprolactinemia immediate after transsphenoidal surgery for lactotroph PitNET presents good predictive value for endocrinological remission without unfavorable effects on the restoration of regular menstruation.

## Data Availability

The datasets used and/or analyzed during the current study are available from the corresponding author on reasonable request.
